# Maternal and fetal prognosis of subsequent pregnancy in black African women with peripartum cardiomyopathy

**DOI:** 10.1186/s12872-018-0856-7

**Published:** 2018-06-18

**Authors:** Nobila Valentin Yaméogo, André Koudnoaga Samadoulougou, Larissa Justine Kagambèga, Koudougou Jonas Kologo, Georges Rosario Christian Millogo, Anna Thiam, Charles Guenancia, Patrice Zansonré

**Affiliations:** 1 Department of Cardiology, Yalgado Ouedraogo University Hospital, Ouagadougou, Burkina Faso; 2 Medical Sciences Department, University of Ouagadougou, Ouagadougou, Burkina Faso; 3 Department of Cardiology, University Hospital, 14 rue Paul Gaffarel, 21079 Dijon CEDEX, France; 4 PEC2, UFR Sciences de Santé, University Bourgogne Franche-Comté, Dijon, France

**Keywords:** Peripartum cardiomyopathy, Subsequent pregnancy, Prognosis, Burkina Faso

## Abstract

**Background:**

The aim of this study was to describe maternal and fetal outcomes after pregnancy complicated by peripartum cardiomyopathy (PPCM).

**Methods:**

We included women that had subsequent pregnancy (SSP) after PPCM and assessed maternal prognosis and pregnancy outcomes, in-hospital up to one week after discharge. Clinical and echocardiographic data were collected comparing alive and deceased women. Factors associated with pregnancy outcomes were assessed.

**Results:**

Twenty-nine patients were included, with a mean age of 26.7 ± 4.6 years and a mean gravidity number of 2.3 ± 0.5 of. At the last medical control before subsequent pregnancy, there was no congestive heart failure, the mean left ventricular diastolic diameter (LVDD) was 53 ± 4 mm and the left ventricular ejection fraction (LVEF) was ≥50% in 13 cases (44.8%).

Maternal outcomes were marked by 14 deaths (48.3%). Among the factors tested in univariate analysis, LVEF at admission had an excellent receiver-operating characteristic (ROC) curve to predict maternal mortality (AUC = 0.95; 95% CI 0.87–1, *p* < 0.001), with a cut off value of < 40% (sensitivity = 93% and specificity = 87%). Concerning fetal outcomes, baseline LVEF had the best area under the curve (AUC) to predict abortion or prematurity among all variables (AUC = 0.75; 95% CI 0.58–092, *p* = 0.003), with a cut-off value of < 50% (sensitivity = 79%, specificity = 67%).

**Conclusions:**

SSP outcomes are still severe in our practice. Maternal mortality remains high and is linked to ventricular systolic function at admission (due to pregnancy), while fetal outcomes are linked to baseline LVEF before pregnancy.

## Background

Peripartum cardiomyopathy (PPCM) is a rare form of heart failure of unknown etiology that occurs between the last month of pregnancy up to 6 months postpartum [1–3]. Although previous studies suggested that approximately 50% of patients with PPCM recover a normal cardiac function, with 25% having persistently reduced heart function stable on medications and 25% progressing to severe heart failure, more recent research [4, 5] suggests that severe outcomes have been reduced, with survival rates as high as 90 to 95% thanks to contemporary medical and device therapy. The Burkina Faso PPCM registry has been prospectively collecting data from women suffering from PPCM since 2010, and estimates the incidence of PPCM around 1/3800 births. According to this registry, even though contraception is prescribed to PPCM women, we noted that some of them get pregnant again.. However, there is a concern that such pregnancy may be associated with an increased risk of recurrence of cardiomyopathy [1–3, 6].

The objective of this study was to determine the incidences and the associated factors of maternal and fetal outcomes of first subsequent pregnancy (SSP) among women with a history of peripartum cardiomyopathy.

## Methods

### Study population

The Burkina-Faso registry of PPCM prospectively includes women diagnosed with PPCM in the university hospital of Ouagadougou and Saint Camille hospital of Ouagadougou. The criteria for the diagnosis of the initial peripartum cardiomyopathy include the development of congestive heart failure within 1 month before delivery to 5 months after delivery in the absence of any another identifiable cause of heart failure; and evidence of depressed left ventricular function, defined as a left ventricular ejection fraction (LVEF) of less than 45% (Simpson method), as measured by echocardiography [7]. Women from the PPCM registry who were hospitalized during a subsequent pregnancy (despite medical advice not to become pregnant after PPCM) were prospectively included from 2012 to 2016. In-hospital to one week post discharge data were collected. We assessed clinical condition, left ventricular diameters and LVEF, tricuspid annular plan systolic excursion (TAPSE) and systolic pulmonary arterial pressure. Delta (%) values of these parameters were assessed as follows: (value at admission – baseline value) / baseline value. Maternal and fetal mortality, pregnancy issues, way of delivery and newborn weight were also appreciated. We compared alive and deceased women to identify the factors associated with maternal death. We also conducted a second analysis focused on fetal prognosis to identify factors associated with miscarriage or prematurity in these women.

### Echocardiographic data collection

- Baseline echocardiographic data are the data from the last cardiac echography before subsequent pregnancy (SSP). This echography was performed on average 2.6 months before pregnancy.
- Admission echocardiographic data come from echography performed on admission during or after SSP.
- During hospitalization, echocardiography was performed weekly until one week post discharge.

### Statistical methods

Continuous variables are presented as means ± standard deviations (SD) when normally distributed or medians and ranges otherwise. Categorical variables are presented as numbers (percentages). For continuous data, normality was checked by the Kolmogorov–test. The characteristics of the deceased and alive groups were compared using the exact Mann-Whitney test for continuous variables and the Chi-square or Fisher’s exact test for categorical variables as appropriate. All of the tests were two-sided, and a *p* value less than 0.05 was considered significant.

To examine determinants of death events, we examined the area under the receiver-operating characteristic (ROC) curve (plot of sensitivity versus 1 − specificity for all possible cut-off values for classifying predictions) for LVEF, systolic Pulmonary arterial pressure (sPAP) and TAPSE with the best sensitivity and specificity according to the Youden index [8]. The cut-off value is given in the results section. Areas under the ROC curves were compared using the method of DeLong et al. [9] for paired data.

All analyses were performed using SPSS 20.0 0 (SPSS, Inc., Chicago, IL, USA) and MedCalc 13.3.1 (MedCalc Softaware, Mariakerke, Belgium).

## Results

### Patient characteristics

During the inclusion period, 29 women followed in the PPCM registry were hospitalized while pursuing a subsequent pregnancy. The mean age was 26.7 ± 4.6 years, with predominance of low socio-economic status. The diagnosis of cardiomyopathy had been made before delivery in 4 women (all of them in the seventh months of pregnancy), during the first month after delivery in 20 women, and between two and six months after delivery in 5 women (3 women in the second month after delivery, 1 in the third month, and 1 in the fifth month). No case of preeclampsia, chronic hypertension, severe anemia or history of hyperthyroidism was identified. Clinical and echocardiographic characteristics of patients during subsequent pregnancy in both groups alive vs deceased (*according to the occurrence of maternal death during subsequent pregnancy*) are summarized in Table 1.

**Table 1:** Clinical and echocardiographic characteristics according to the occurrence of death during subsequent pregnancy after PPCM

n (%), median (interquartile range), mean ± SD	Total population	Alive patients (*n* = 15)	Deceased patients (*n* = 14)	p
Baseline data				
Age	26.7 ± 4.6	26 ± 5	28 ± 6	0.44
Low socio-economic status	22 (75.8)	12 (80)	9 (64)	0.43
Gravidity	2.3 ± 0.5	2.2 ± 0.6	2.4 ± 0.5	0.26
Baseline LVEF after the first PPCM (%)	49.9 ± 5.2	50 ± 6	50 ± 7	0.99
Baseline LVEF after the PPCM < 50%	16 (55.2)	7 (47)	9 (64)	0.46
Baseline LVEF after the first PPCM < 45%	5 (17.2)	2 (13)	4 (29)	0.39
Baseline LVEDD after the PPCM (mm)	53.3 ± 3.6	54 ± 4	53 ± 5	0.88
Baseline sPAP after the PPCM (mmHg)	29.2 ± 4.2	28 ± 5	30 ± 5	0.26
Baseline TAPSE after the PPCM (mm)	18.2 ± 2.4	19 ± 3	17 ± 3	0.10
Follow-up data (at the time of hospitalization)				
Delay between last follow-up and hospitalization (month)	17 ± 6	15 ± 6	18 ± 6	0.27
Congestive heart failure	19 (65.5)	6 (40)	14(100)	< 0.001
LVEF (%)	37.6 ± 4.7	44 ± 6	33 ± 6	< 0.001
LVEF < 40%	14 (48.3)	2 (13)	13 (93)	0.001
Delta LVEF (%)	−19.3 ± 9	- 11 ± 10	−32 ± 15	< 0.001
LVEDD (mm)	57.5 ± 3.9	59 ± 4	56 ± 5	0.05
Delta LVEDD (%)	8 ± 6	11 ± 11	5 ± 7	0.09
sPAP (mmHg)	37.9 ± 5.4	34 ± 5	42 ± 5	< 0.001
Delta sPAP (%)	19 ± 8	17 ± 10	27 ± 15	0.07
TAPSE (mm)	16.8 ± 3.2	19 ± 3	14 ± 4	< 0.001
TAPSE < 17 mm	11 (37.9)	1 (7)	10 (71)	< 0.001
TAPSE < 18 mm	13 (44.8)	2 (13)	11 (79)	< 0.001
Delta TAPSE (%)	- 7.5 ± 5	3 ± 17	−15 ± 27	0.03
Pregnancy data				
Abortion	6 (20.6)	2 (13)	4 (29)	0.39
Prematurity	8 (27.6)	4 (27)	4 (29)	1
Full-term pregnancy	51.7	9 (60)	6 (43)	0.47
Newborn weight (g)	2385 ± 286	2350 ± 500	2380 ± 300	0.90

*PPCM* peripartum cardiomyopathy, *LVEF* left ventricular ejection fraction, *LVEDD* left ventricular end diastolic diameter, *sPAP* systolic pulmonary arterial pressure, *TAPSE* tricuspid annular plan systolic excursion

### Maternal outcomes

The mean LVEF in the total cohort of 29 women at the last follow-up before pregnancy was 49.9 ± 5.1% and decreased significantly to 37.6 ± 4.7% after the first subsequent pregnancy with a mean delta LVEF of − 19.3 ± 9%.

Fourteen (14) women died (48.3%). There was no statistically significant difference between alive and deceased women concerning socioeconomic status, left ventricular and right ventricular systolic function before subsequent pregnancy (baseline). At the time of hospitalization (during the subsequent pregnancy) (Table 1), congestive heart failure (*p* < 0.001), with lower left and right ventricular function was more frequently observed in women who died after pregnancy than in the group of surviving patients.

**Fig. 1:**
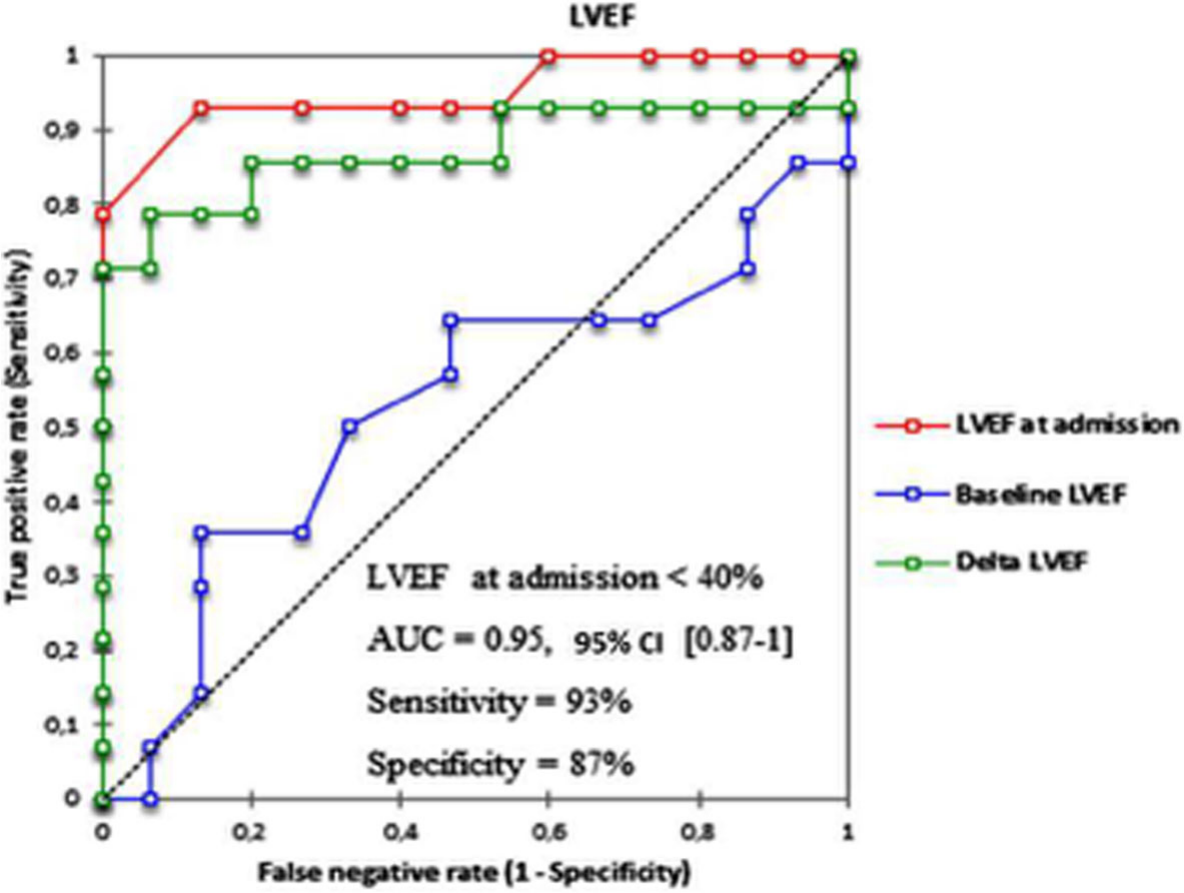
ROC curves with optimal cut-off values of LVEF (admission, baseline and delta) to predict mortality

**Fig. 2:**
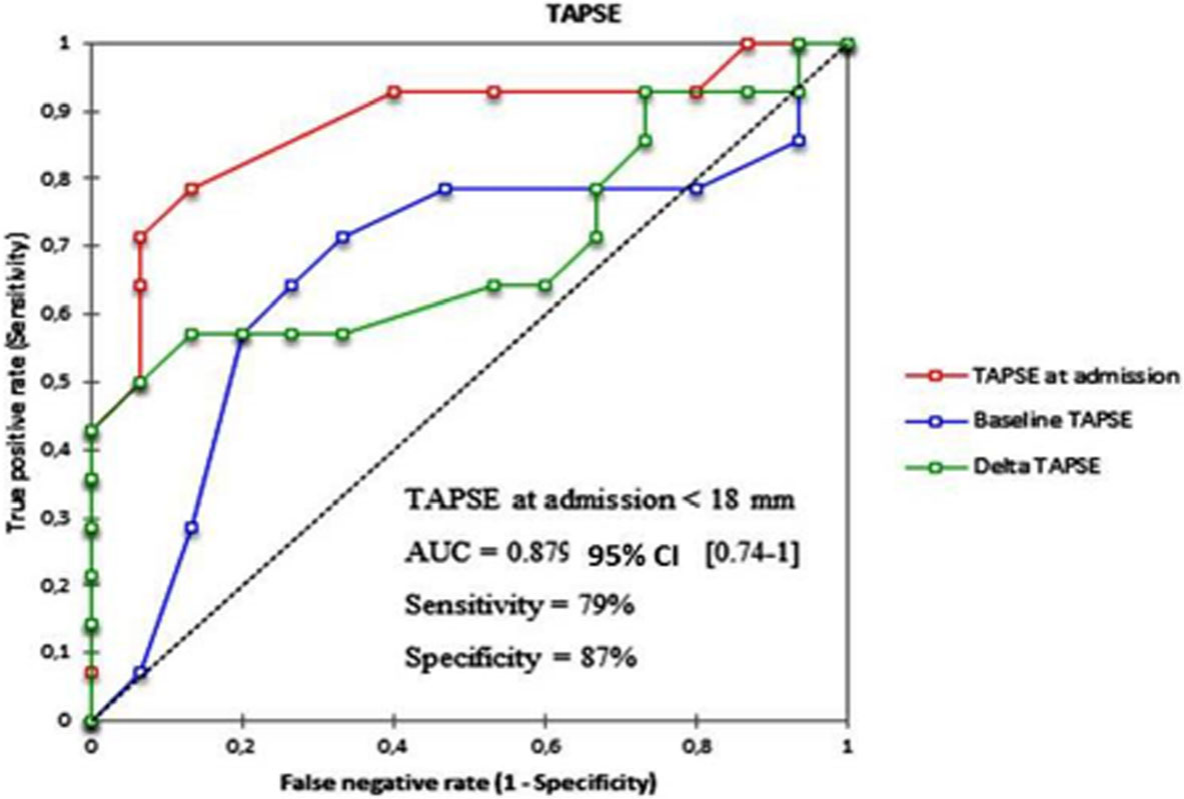
ROC curves of TAPSE (admission, baseline and delta) to predict mortality

**Fig. 3:**
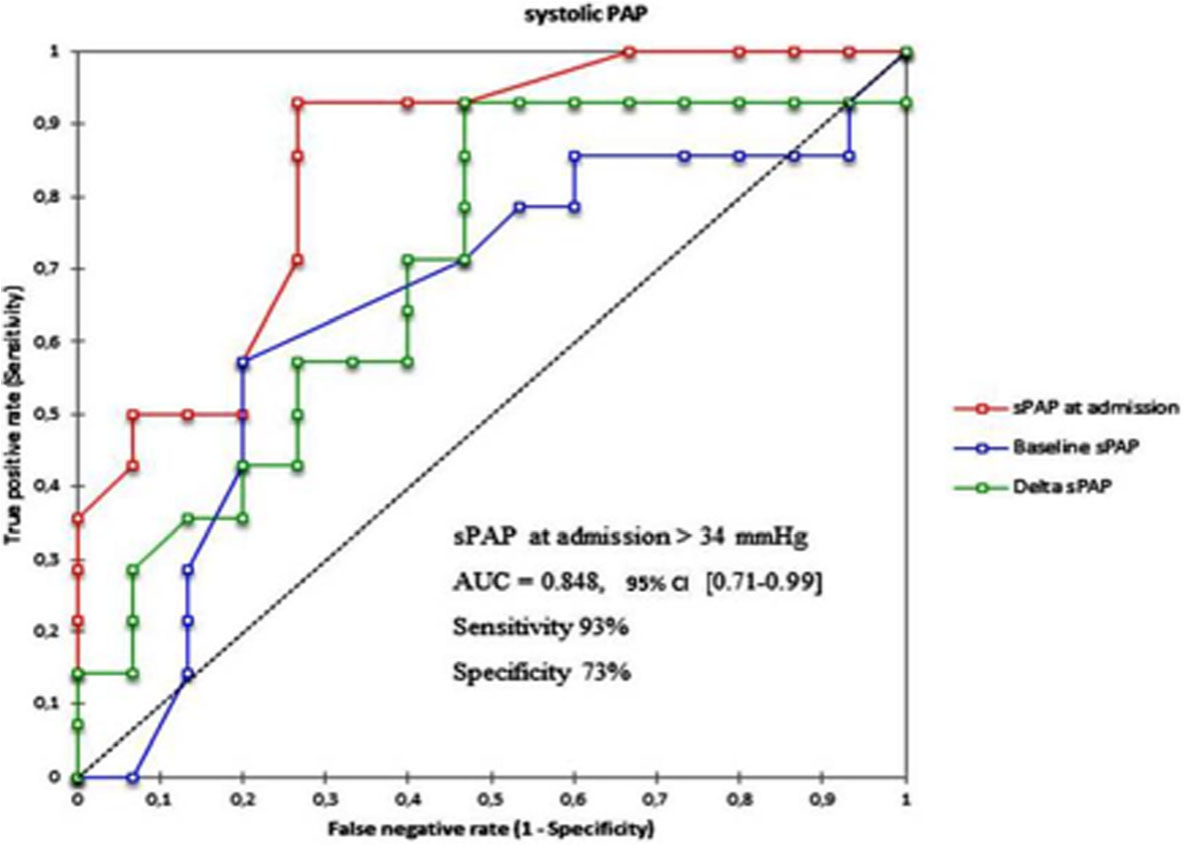
ROC curves of sPAP (admission, baseline and delta) to predict mortality

To find out if echocardiographic data (at the last follow-up and at admission) could predict mortality after pregnancy, we built ROC curves using variables with a strong statistical association to death in univariate analysis: LVEF (at last follow-up (=baseline), at admission and delta LVEF), sPAP (at last follow-up, at admission and delta sPAP), and TAPSE (at last follow-up, at admission and delta TAPSE). The AUC were compared to determine the best variable, and the best cut-off value. Among these variables, LVEF at admission (AUC = 0.95; 95% CI 0.87–1, *p* < 0.001), TAPSE at admission (AUC = 0.879; 95% CI 0.74–1, *p* = 0.001) and sPAP at admission (AUC = 0.85; 95% CI 0.71–0.99, p = 0.001) were strongly and significantly associated to death (Figs. 1, 2, 3). Comparing the AUC of these 3 variables at the time of admission (data not shown), there was no statistically significant difference. The best cut-offs for these variables are detailed within the figures.

### Pregnancies outcomes

Miscarriage occurred in six cases. Among the 23 women with a living child after subsequent pregnancy, 13 had a normal vaginal delivery, and 10 were delivered by cesarean. The mean newborn weight was 2385 ± 286 g. Miscarriage and prematurity were associated with baseline LVEF and baseline TAPSE as shown in Table 1. Baseline LVEF had the best AUC to predict SSP outcomes among all variables (AUC = 0.75; 95% CI 0.58–092, *p* = 0.003), with a cut-off value of < 50% (sensitivity = 79%, specificity = 67%).Four newborn (that 3 preterm) died, all from neonatal infection.

## Discussion

### Maternal outcomes

Our study demonstrated that when women with SSP did not benefit from cardiac follow-up, the issue can be fatal.

Mortality was remarkably high in our study, probably because the women were lost from clinical follow-up during the pregnancy period.

In several studies [10–12], SSP was responsible for maternal death in about 0.5 to 1%. This contrasts with our results. This can be explained by the fact that our patients were lost from follow-up and the lack of therapeutic means. Indeed follow-up enables an effective management of heart failure, pregnancy and delivery. During this follow-up, normalization of left ventricular function does not guarantee an uncomplicated SSP as demonstrated by some authors in America and Europe. In their study, approximately 20% of such patients are also at risk of moderate to severe deterioration of left ventricular function, which persists after delivery in 20 to 50% of cases [10–12].

We demonstrated that left ventricular ejection fraction decreased during SSP.

### Factors associated with maternal death

The best determinant of maternal death in our study was the left ventricular ejection fraction at admission and the percentage of loss of left ventricular ejection fraction. The best cut-off value of left ventricular ejection fraction to predict mortality during first SSP in our study was a LVEF at admission < 40% [Sensitivity = 93%, Specificity = 87%].

All previous studies were based on left ventricular function, but right ventricular function was not studied. We demonstrate that right ventricle systolic function also has an important role in the prediction of maternal mortality during SSP. We appreciated right ventricular systolic function by TAPSE, but we also measured systolic pulmonary arterial pressure to determine if sPAP could predict mortality during SSP.

We found that all these parameters were associated to maternal death. These results underline that right ventricular function must be taken into account to estimate maternal outcomes of SSP. In a study by Elkayam et al. [10], medical pregnancy interruption was used in some cases to prevent maternal outcomes. In their study, maternal mortality was very low compared to our result, but medical pregnancy interruption was more frequent.

All our results demonstrate that PPCM women with SSP are very fragile. They must benefit from a rigorous follow-up including echocardiographic assessment.

### Pregnancy outcomes and infant morbi-mortality

Pregnancy outcomes were also poor. We noted that miscarriage and prematurity were associated with baseline left ventricular ejection fraction and baseline TAPSE. This can be explained by the fact that low baseline left ventricular ejection fraction compromises fetal development. The same reason can explain why our new borns had all small birth weight.

Elkayam et al. [10] demonstrated that abortion rate is very high (20.4%), and prematurity is two times more frequent in women with persistent baseline left ventricular dysfunction. In our study, not only baseline left ventricular ejection, but also baseline TAPSE was responsible for pregnancy outcomes.

### Infant mortality

Death occurred predominately in premature new borns (3/4). There was no maternal nor fetal factor linked to these deaths. However, as we know, prematurity is a factor of mortality in new born babies.

### Limitations

Our study included only a small number of PPCM women but it is to the best of our knowledge the biggest African study on SSP outcomes.

A recruitment bias has to be acknowledged: only hospitalized women were included, due to loss of follow-Up. Indeed, from the 166 women included in the registry, 38 were lost to follow-up. From these lost to follow-up women, 29 were hospitalized during a SSP and 9 were missing. None of the 128 patients followed in the registry had so far a SSP. Among the 9 patients missing, uncomplicated pregnancies could have occurred. Thus, the mortality rate of SSP described in our study could be slightly overestimated.

## Conclusions

Some women with a history of PPCM hope to get pregnant again. They are therefore at risk because pregnancy outcomes are unknown.

This study demonstrated that subsequent pregnancy after peripartum cardiomyopathy has poor maternal and fetal prognosis.

Maternal mortality is very high and associated with LVEF at admission, TAPSE at admission and sPAP at admission. On the other hand, pregnancy outcomes were linked to baseline LVEF and baseline TAPSE.

In these conditions, we advise physicians, to systematically explain to PPCM patients the severity of their pathology, the possibility of evolution and to give them a drafted document that contains recommendations in this situation (subsequent pregnancy risk, medical conditions to get pregnant, necessity to continue follow-up).

## Abbreviations

AUC: Area under the curve
LVDD: Left ventricular diastolic diameter
LVEF: Left Ventricular Ejection Fraction
PPCM: Peripartum cardiomyopathy
ROC: Receiver-Operating Characteristic
SD: Standard deviations
sPAP: Systolic pulmonary arterial pressure
SSP: Subsequent pregnancy
TAPSE: Tricuspid annular plan systolic excursion
